# Real-Time Monitoring of School Absenteeism to Enhance Disease Surveillance: A Pilot Study of a Mobile Electronic Reporting System

**DOI:** 10.2196/mhealth.3114

**Published:** 2014-05-12

**Authors:** Saranath Lawpoolsri, Amnat Khamsiriwatchara, Wongwat Liulark, Komchaluch Taweeseneepitch, Aumnuyphan Sangvichean, Wiraporn Thongprarong, Jaranit Kaewkungwal, Pratap Singhasivanon

**Affiliations:** ^1^Department of Tropical HygieneFaculty of Tropical MedicineMahidol UniversityBangkokThailand; ^2^Center of Excellence for Biomedical and Public Health Informatics (BIOPHICS)Faculty of Tropical MedicineMahidol UniversityBangkokThailand; ^3^Center for Emerging and Neglected Infectious DiseasesMahidol UniversityBangkokThailand; ^4^Communicable Disease Control DivisionDepartment of HealthBangkok Metropolitan AdministrationBangkokThailand

**Keywords:** syndromic surveillance, schools, absenteeism, tablets, reporting system

## Abstract

**Background:**

School absenteeism is a common source of data used in syndromic surveillance, which can eventually be used for early outbreak detection. However, the absenteeism reporting system in most schools, especially in developing countries, relies on a paper-based method that limits its use for disease surveillance or outbreak detection.

**Objective:**

The objective of this study was to develop an electronic real-time reporting system on school absenteeism for syndromic surveillance.

**Methods:**

An electronic (Web-based) school absenteeism reporting system was developed to embed it within the normal routine process of absenteeism reporting. This electronic system allowed teachers to update students' attendance status via mobile tablets. The data from all classes and schools were then automatically sent to a centralized database for further analysis and presentation, and for monitoring temporal and spatial patterns of absent students. In addition, the system also had a disease investigation module, which provided a link between absenteeism data from schools and local health centers, to investigate causes of fever among sick students.

**Results:**

The electronic school absenteeism reporting system was implemented in 7 primary schools in Bangkok, Thailand, with total participation of approximately 5000 students. During May-October 2012 (first semester), the percentage of absentees varied between 1% and 10%. The peak of school absenteeism (sick leave) was observed between July and September 2012, which coincided with the peak of dengue cases in children aged 6-12 years being reported to the disease surveillance system.

**Conclusions:**

The timeliness of a reporting system is a critical function in any surveillance system. Web-based application and mobile technology can potentially enhance the use of school absenteeism data for syndromic surveillance and outbreak detection. This study presents the factors that determine the implementation success of this reporting system.

## Introduction

Disease epidemics occur easily now due to rapid transportation and increased social contact. Traditional disease surveillance that is based only on disease reporting systems may not be sufficient for timely detection of a disease epidemic. There is growing interest in applying syndromic surveillance to enhance outbreak detection capabilities. Unlike traditional surveillance, syndromic surveillance uses prediagnostic clinical data such as emergency department visits, laboratory ordering volume, and other surrogate data indicating early illness, such as school or work absenteeism and over-the-counter medication sales. Using these prediagnostic data can potentially improve timeliness in outbreak detection [1]. Syndromic surveillance systems are widely utilized; more than 80% of public health agencies in the United States and Canada have been using syndromic surveillance [2-4].

School absenteeism is a common data source used in syndromic surveillance [2-4]. Schoolchildren are at high risk for many infectious diseases and share common risk factors at school. Infectious diseases are likely to spread easily in schools due to frequent contact among students. Schoolchildren play an important role in disease transmission in the community, because of their contact patterns with adults and preschool children at home [5]. Monitoring school absenteeism, therefore, can be a useful tool for predicting disease outbreaks. The usefulness of school absenteeism data in predicting outbreaks was widely documented during the influenza H1N1 epidemic in 2009 [6-8]. However, timeliness of data is the most important requirement for using school absenteeism for syndromic surveillance. An electronic school absenteeism reporting system is required for real-time or near real-time monitoring of the absentees; this could limit the use of school absenteeism data in developing countries.

In Thailand, continuous monitoring of school absentees has not been applied in a disease surveillance system. Traditional school absentee reports rely on a paper-based system. Every morning, teachers are responsible for reporting the number of absentees in each class to the school registration unit where a daily summary report on absenteeism is manually generated and stored. Daily data on school absenteeism were reviewed and used only by individual schools. Summary reports are usually submitted to the centralized level (Department of Education) once a semester for financial and human resources consideration purposes. Data sharing between the Department of Education and Department of Health is very limited based on this traditional system. This study aimed to develop a real-time school absenteeism reporting system that also can be used for syndromic surveillance in Thailand.

## Methods

### Study Area

**Figure 1:**
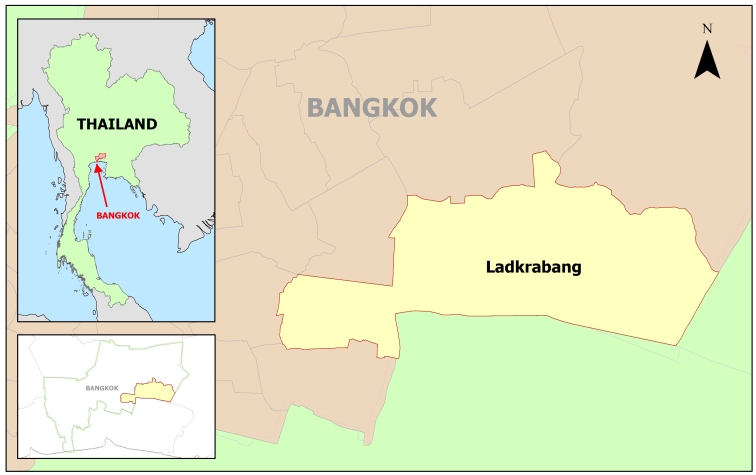
Study area (highlighted area), Ladkrabang district of Bangkok, Thailand.

The study was conducted in primary schools under the Bangkok Metropolitan Administration (BMA), in Ladkrabang district, Bangkok, Thailand (Figure 1). Seven of 20 schools under the BMA were stratified by school size and randomly selected to represent all schools with different school sizes in the area. The number of students ranged from 100 to 2000 students per school, with a total of 5000 students. The basic characteristics of the 7 participating schools are shown in Table 1.

**Table 1:** Characteristics of the 7 participating schools.

School	No. students	Average class size	No. teachers	No. staff	Median percent of absence (range)	Median percent of sickness with fever (range)
1	1970	36	89	17	7 (0-91)^a^	0 (0-2)
2	238	30	11	9	7 (0-66)^a^	0 (0-3)
3	456	38	24	10	5 (0-51)^a^	1 (0-3)
4	636	35	37	5	3 (0-8)	0 (0-4)
5	633	35	30	13	3 (0-9)	0 (0-1.3)
6	813	39	44	18	6 (0-63)^a^	2 (0-14)
7	85	14	10	6	4 (0-29)^a^	0 (0-6)

^a^The extremely high absence rates occurred during examination week.

### The Reporting System

**Figure 2:**
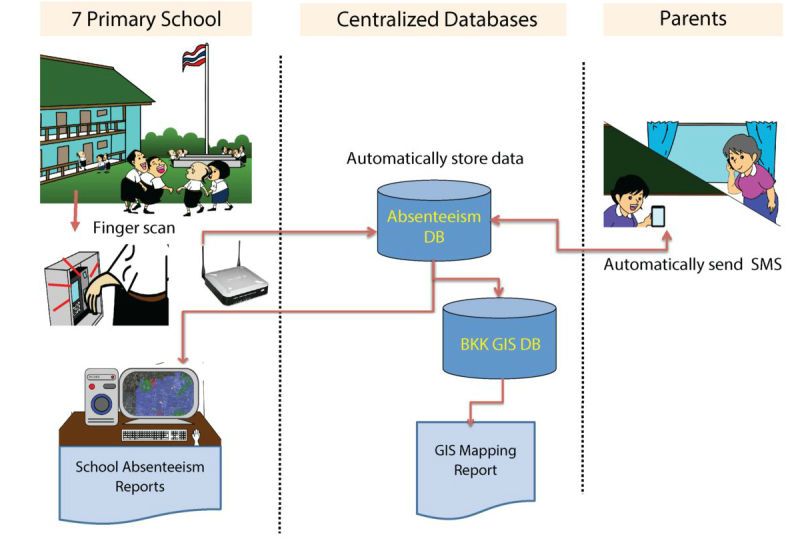
Initial data and work flow of the school absenteeism reporting system.

An electronic school absenteeism reporting system was developed and implemented in 7 schools in 2011. The system was designed to use fingerprint scans as a measure of school attendance. Schoolteachers, parents, and students were informed of the new absenteeism reporting system, and students were required to provide fingerprint scans every morning when they arrived at the school. Attendance data from fingerprint scans from all 7 schools were then automatically sent to the centralized database, which is linked to the geographical information system database where data on the students’ house location is stored. In addition, text messaging (short message service) function was integrated into the system so that a text message was automatically generated and sent to the parent of that specific absent individual. To increase the specificity of absenteeism data in predicting disease outbreak, parents were contacted by phone and asked for the reason for the student’s absence, and if the student was sick, if he or she had a fever. A summary of initial work and data flow is shown in Figure 2.

After the implementation of the fingerprint scan system, potential problems were observed. A significant number of students failed to scan each day, an approximate 20% error rate, thereby requiring confirmation of school absenteeism data from the teachers; the assigned teachers had to submit confirmed absenteeism data through the Web-based system. A negative response was received after the system was launched due to the extra workload for schoolteachers.

**Figure 3:**
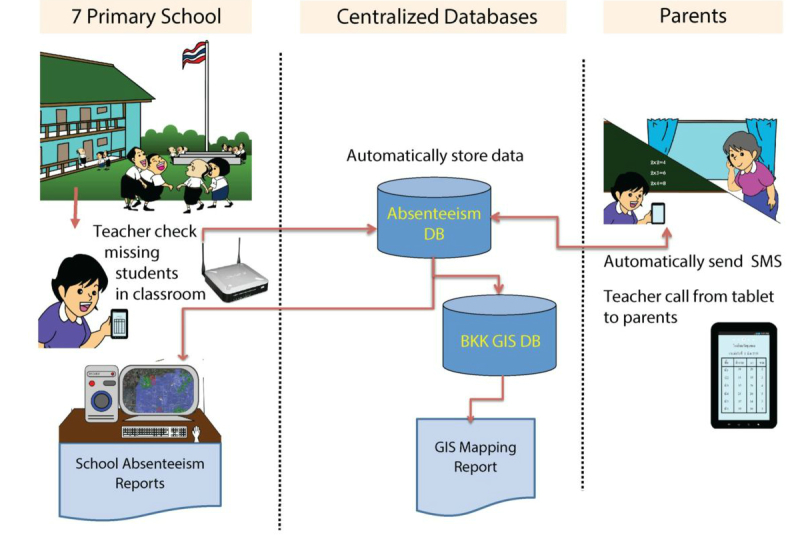
Revised data and work flow of the school absenteeism reporting system.

The school absenteeism reporting system was then revised to embed the electronic reporting system into the routine process of absenteeism reporting (Figure 3). The traditional absenteeism reporting system required teachers to record absent student data onto a standard form (paper-based). To continue this routine, an electronic version of the absenteeism report form was created, and teachers could access this electronic form through a Web-based system. The parent’s phone numbers were obtained from the electronic student registration system, which is an existing system created and used by the Department of Education.

**Figure 4:**
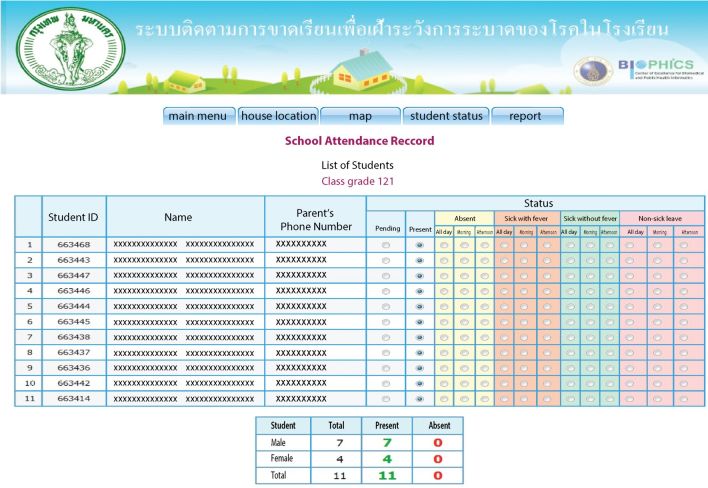
Web page for entering data of the school absenteeism reporting system.

**Figure 5:**
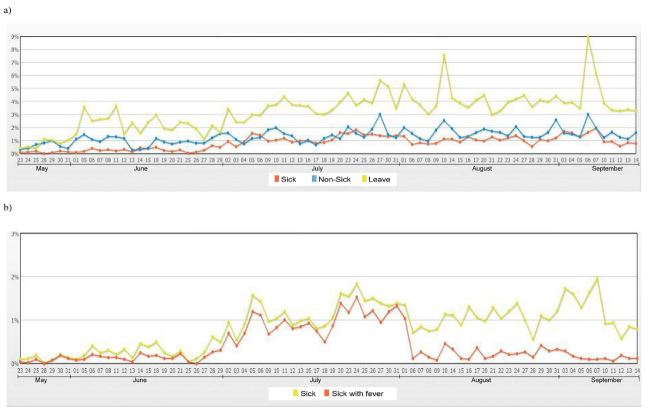
Graphs showing patterns of school absenteeism generated from the system from 24 May to 14 September 2012 (First Semester). a) Pattern of overall absenteeism; b) Pattern of sick leave with and without fever.

To assist in the data entering process, tablet computers with Internet and phone connection were distributed to teachers. Teachers could update the school attendance status of the children in their classes immediately and the data were then automatically sent to a centralized database. The Web-based reporting system also provided the parent’s phone number, which allowed teachers to call parents directly from the tablets to ask for the reasons for school absenteeism (Figure 4). In addition, a standard school absenteeism summary form could be automatically generated. This summary form was designed to look like the existing form that is submitted to the Department of Education once a semester. With this Web-based reporting system, data on school absenteeism, including reasons for absence in the 7 schools would be updated to the centralized database on a real-time or near real-time basis. Authorized persons from the Department of Education and Department of Health could view these real-time data via the Web-based system (Figure 5). Informative statistics such as graphs of number and percent of school absentees were generated to present patterns of school absenteeism over specific periods. After implementation of this revised system, teachers were very satisfied with this system, because the system could potentially reduce their workload and they can appreciate the usefulness of the system.

### The Disease Investigation Module

**Figure 6:**
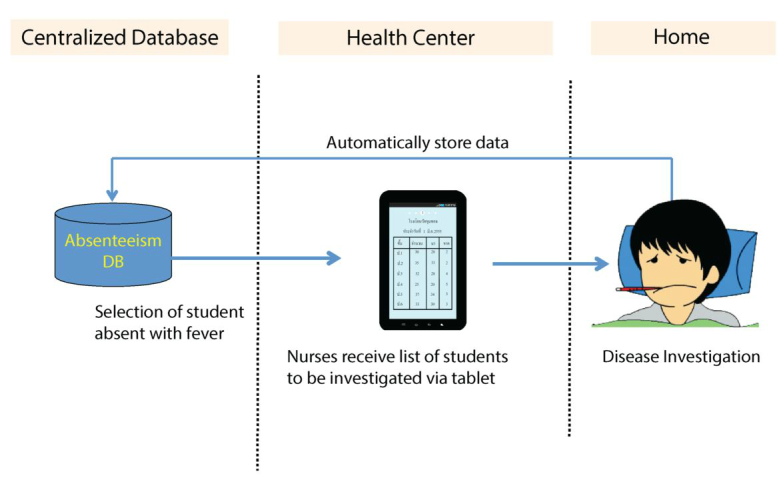
Data and work flow of the disease investigation module.

Disease investigation is an important function in disease surveillance, especially during an outbreak. Communication between schools and health care sectors plays a critical role in disease investigation, especially when there is a suspected outbreak among schoolchildren. Therefore, an additional module that provides a link between school and local health care centers has been developed (Figure 6). In this disease investigation module, the system allows absent students with fever to be recognized at local health care centers via the Web-based application. In addition, health care personnel who are responsible for disease investigation can receive information about the sick student, including address and contact phone number via the mobile tablets. This module also allows health care personnel to collect results of their disease investigation through the tablet, and that data can then automatically be sent to the centralized database. This disease investigation module was implemented in October 2013.

## Results

The school absenteeism reporting system was fully functional in May 2012 (first semester of academic year 2012). The data on school absentees and their reasons for absence were updated every morning of each school day. At the beginning, teachers were not accustomed to the new routine and therefore staff members that monitored the centralized database would call or send a message to the teacher to remind him or her to submit the absenteeism information. The number of missing data significantly decreased over time. After the first few months of the operation, the completeness of absenteeism data was comparable with that of the original paper-based reporting system. Although the data on causes for the absence were not required in the original paper-based system, in the new electronic reporting system, teachers were asked to contact parents of absent students regarding the cause for the absence. If the first contact was unsuccessful, follow-up calls would be made. Overall, the percent of successful contact with parents has increased from approximately 70% in the first few months of operation to approximately 90% after 1 year of implementation.

**Figure 7:**
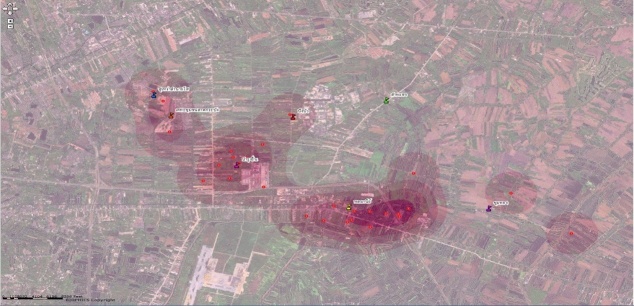
Clusters of school absentees, generated by the school absenteeism reporting system.

Our results show that of 5000 students in 7 schools, approximately 1%-10% of students were absent each day. The absence rate also varied across schools (Table 1). When school absenteeism was classified according to the reason for the absence, the number of sick students ranged from 10 to 100 per day. Among sick students, approximately half of them had a fever. The percentage of student with sickness and fever varied, ranging from 0% to 2% (median = 1%). The pattern of absence rate and percent of sickness stratified by schools are illustrated in Multimedia Appendices 1 and 2. The system also provided a function to query the number of children who had fever for 2 or more consecutive days, which is more specific for diseases with prolonged fever such as dengue infection, an endemic disease in the area. The number of school absentees who were sick with fever for 2 or more consecutive days ranged from 1 to 28 (median = 6) students per day, with the percentage ranging from 0% to 0.3% (median 0.1%). In addition, the house location of absentees could be observed from a map link, and clusters of school absentees were also detected by using the point density analysis with the spatial analysis tool in ArcMap (Figure 7). This geographical information system function can enable early warning of a possible disease outbreak in a community. Although the clusters of school absentees in this map may be biased due to a large number of students living near the large school, this geographical information system function would be more beneficial when the system is expanded to cover more schools distributed across the area.

**Figure 8:**
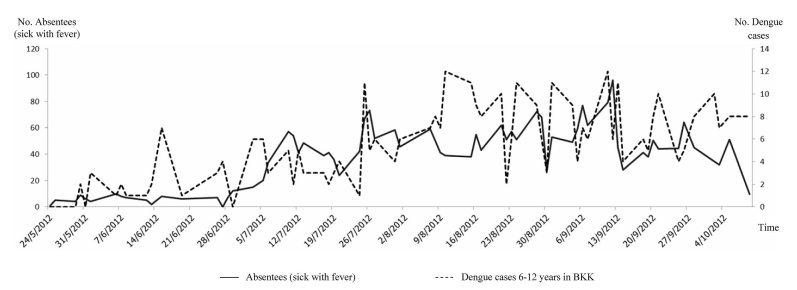
Patterns of daily number of school absentees (sick leave) and daily number of dengue cases during May 24 - October 9, 2012 (first semester).

**Figure 9:**
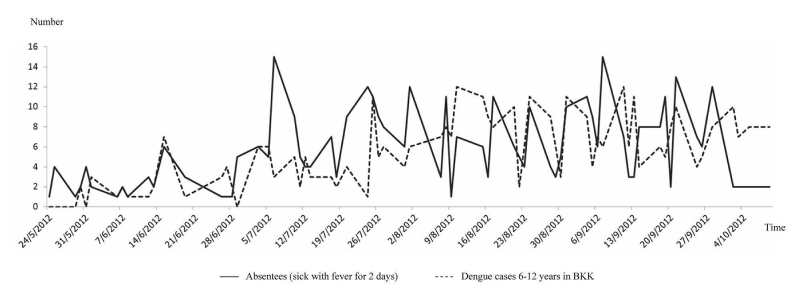
Patterns of daily number of schoolchildren who had fever for 2 consecutive days and daily number of dengue cases during May 24 - October 9, 2012 (first semester).

School absenteeism also varied over the course of the year; during May-October 2012 (first semester), school absenteeism (sick with fever) peaked between July and September 2012. This period coincided with a peak of dengue cases in children 6-12 years old in Bangkok, according to the BMA surveillance system, with a correlation coefficient of .57 (Figure 8). The correlation with dengue cases was also observed when compared with the number of school absentees who were sick with fever for 2 consecutive days, with a correlation coefficient of .32. The first peak of dengue started in mid-June, then declined and rose again in early July, and then persisted until October; the pattern was consistent with the absenteeism pattern observed among students in the 7 schools (Figure 9). To explore the performance of the system for the early detection of dengue cases, the number of dengue cases at the day report received and number of school absentees who were sick with fever at each day with different lag times were calculated. The correlation was stronger with lag times of 14 days (*R* ^2^=.57, .54, and .61 for a lag time of 0, 7, and 14 days), in which the case report in the existing passive surveillance system is usually delayed approximately 2 weeks.

## Discussion

School absenteeism data are among the most frequently used sources for a syndromic surveillance system [3]. However, timeliness of data is a key factor in using school absenteeism data for outbreak prediction. This study demonstrated the application of information technology to improve school absenteeism reporting systems. Electronic data entry via mobile devices and a Web-based reporting system was designed to replace the traditional paper-based school absenteeism report. In Thailand, school absenteeism data have not been routinely shared and used for prediction of disease epidemics. In this study, a centralized electronic school absenteeism database was created that can be shared among participating schools, the Department of Education, and the Department of Health. The data can be used not only for syndromic surveillance purposes, but also for educational and social purposes.

A successful health information system can be determined by factors including technical, social, and organizational [9]. A smart card or fingerprint scan can be used as an electronic method to record school attendance [6]. Fingerprint scans instead of smart cards were initially implemented in this study due to a concern about the accuracy of using smart cards; for instance, students may forget to bring their cards to school, or may give their card to friends to represent their attendance at school. After the implementation of fingerprint scans, a significant number of students were observed who failed to scan. Therefore, a Web-based reporting system was used to replace the fingerprint scan to record school attendance. Web-based school absenteeism reporting systems have been implemented in many countries [7,8,10]. In most Web-based reporting systems, all data of school absentees are recorded by teachers and usually input into the database by one or a few teachers who are responsible for this step. According to our study, this procedure did not work well due to the extra work required by teachers. In addition, this procedure was outside their normal routine and could therefore potentially affect the sustainability of the system.

A change in technical procedure outside the normal routine may determine the successfulness of the system. This study suggested that a system designed to follow routine procedures was likely to be a success. In our system, the only change from routine procedure was that teachers had to record data of school absentees into computer tablets, rather than on paper. This system also helped to reduce the school’s administrative office workload by automatically generating a summary of school absenteeism. Computer tablets are now widely available at affordable prices and people have easier access to this kind of technology than in the past. In addition, computer tablets can be used for other purposes besides reporting school absenteeism. Therefore, this would be a worthwhile investment when policymakers want to expand this system to other schools.

Key stakeholders and an organization’s staff perceptions are key factors to the implementation success of information systems; the system should be beneficial to all groups involved. At a national level, the school absenteeism database can be used to assist disease surveillance and outbreak prediction, whereas at the local or school level the system can automatically create absenteeism reports for that particular school. The commitment and perceptions of school principals were also important in system implementation. In this study, the participation of staff in each school depended mostly on the leader’s commitment rather than school size or resources of each school.

Although school absenteeism data has been widely used as an adjunct indicator for outbreak prediction, the lack of specificity of this type of data remains a major concern [3]. A system that relies on school absenteeism data alone without knowing the reasons for absence are less likely to be useful for outbreak prediction [6-8,10,11]. In this study, parents of absentees were contacted by the teacher to ask about reasons for the absence. The reasons were classified as non-sick leave, sick with fever, or sick without fever. In addition, students who were sick with fever for 2 or more days could be simply identified by the system, which would increase the specificity of our system for outbreak prediction. However, more specific symptoms may be added into the system to improve the accuracy of the data.

The main limitation of using school absenteeism data for outbreak prediction is missing information during school breaks. However, the normal epidemic period of common diseases such as dengue and influenza is in the rainy season, between June and August, which falls during the normal school term. In addition, the causes of school absence could be varied; a high absent rate may be due to events such as examination or special events that are not related to health problems. Therefore, for the purpose of disease surveillance, extra care is needed when using school absenteeism data; using an absence rate due to sickness or sickness with fever may be more specific for disease surveillance purposes.

In this study, the peak of school absenteeism coincided with dengue occurrence in Bangkok among 6- to 12-year-old children. Dengue is a common disease in children, especially in a large city. In a cohort study of schoolchildren in a province of Thailand, dengue accounted for almost 7% of children with febrile illness [12]. In this study, the correlation was strongly observed when using absenteeism data of those who had fever for 2 or more days. However, the number of absentees was relatively higher than dengue occurrence, taking into consideration that the school absenteeism data in this study were obtained from only 7 schools in a district of Bangkok. This suggests that there could be diseases other than dengue that contributed to student absenteeism. The investigation of the cause of fever among schoolchildren is ongoing, using the disease investigation module in our system.

Only primary schools under BMA were included in this study. Disease transmission patterns may be different between primary schools and other school levels; however, incidences of important infectious diseases, such as dengue and influenza, are higher among children in primary schools than in secondary schools [13,14]. In addition, only schools under BMA may not be a good representative of all schools in Bangkok, which also include private schools and schools under the Ministry of Education. Therefore, an expansion of the system is planned to cover more schools in other districts of Bangkok that can be used as sentinel sites for syndromic surveillance in the near future.

## Multimedia Appendix 1

Pattern of absence rate, stratified by schools.

## Multimedia Appendix 2

Pattern of percent of sick leave, stratified by schools.

## Abbreviations

BMA: Bangkok metropolitan administration
