# Supplementary figures and images for: Real-Time Monitoring of School Absenteeism to Enhance Disease Surveillance: A Pilot Study of a Mobile Electronic Reporting System
Source: JMIR Mhealth Uhealth. 2014 May 12;2(2):e22. doi: 10.2196/mhealth.3114 (PMC4114464; doi:10.2196/mhealth.3114)

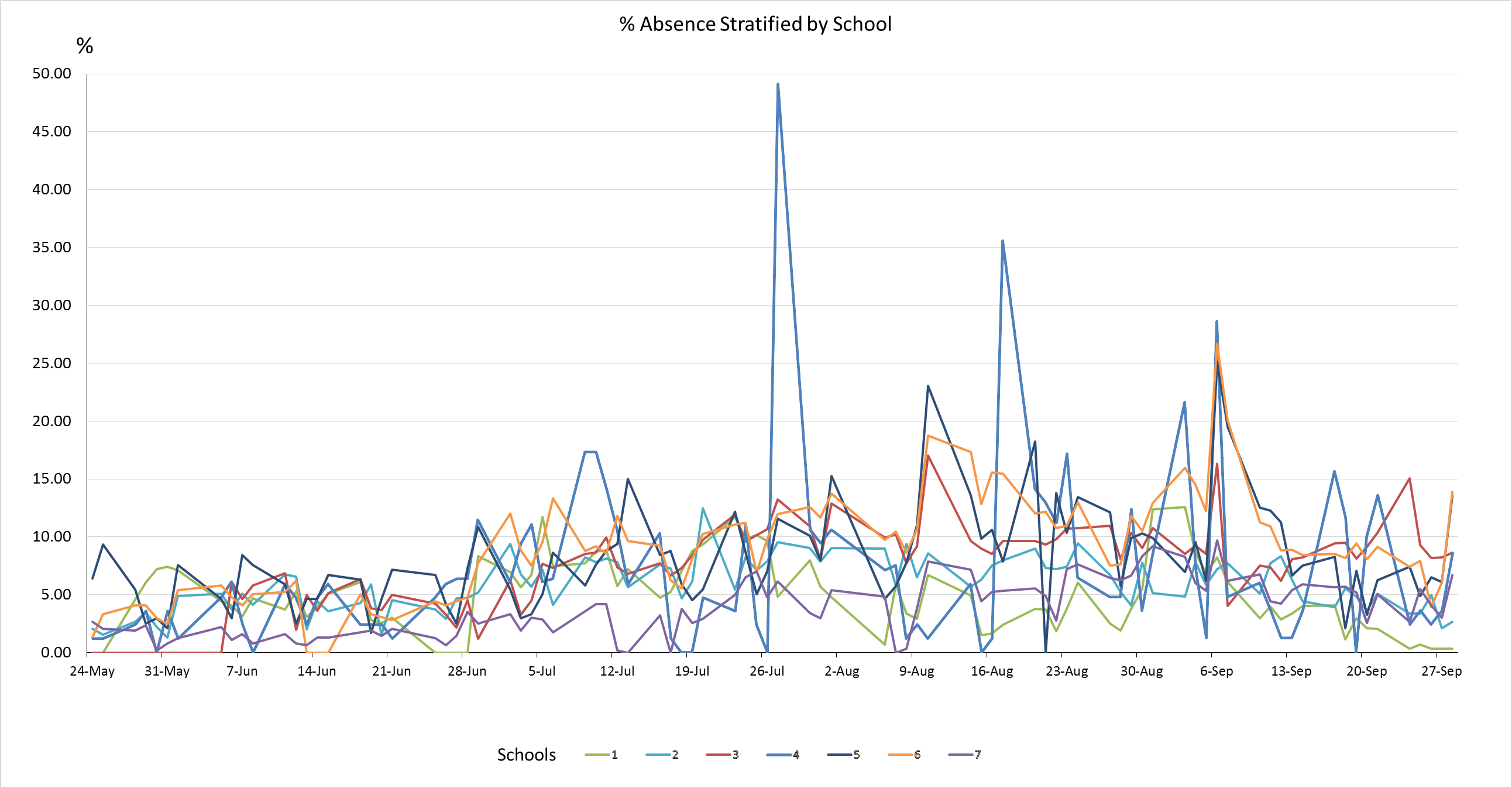

Supplement: Supplementary file 1 [file mhealth_v2i2e22_app1.png]

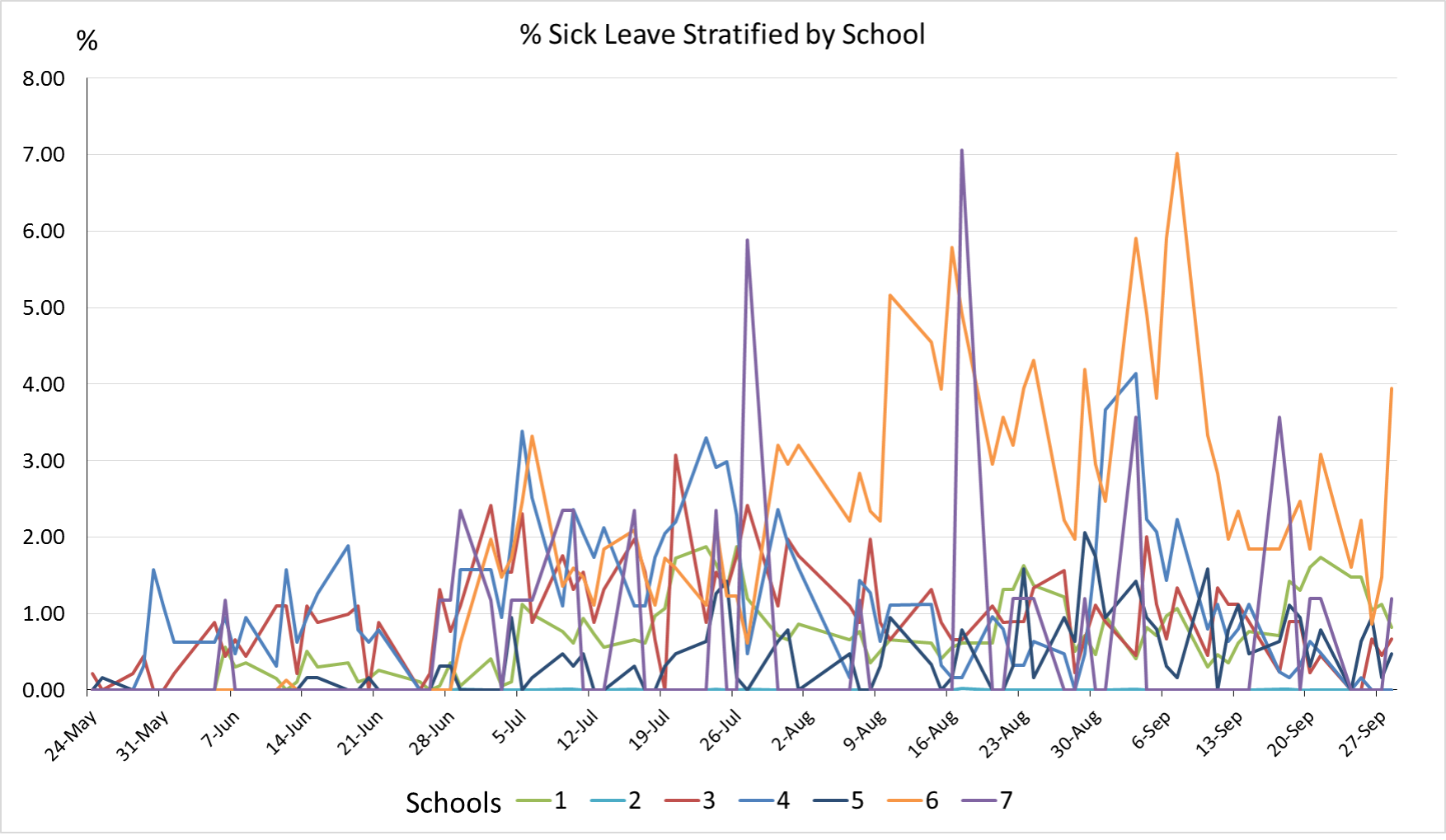

Supplement: Supplementary file 2 [file mhealth_v2i2e22_app2.png]
